# Full-length human dystrophin on human artificial chromosome compensates for mouse dystrophin deficiency in a Duchenne muscular dystrophy mouse model

**DOI:** 10.1038/s41598-023-31481-3

**Published:** 2023-03-16

**Authors:** Yosuke Hiramuki, Satoshi Abe, Narumi Uno, Kanako Kazuki, Shuta Takata, Hitomaru Miyamoto, Haruka Takayama, Kayoko Morimoto, Shoko Takehara, Mitsuhiko Osaki, Jun Tanihata, Shin’ichi Takeda, Kazuma Tomizuka, Mitsuo Oshimura, Yasuhiro Kazuki

**Affiliations:** 1 Chromosome Engineering Research Center, Tottori University, 86 Nishi-cho, Yonago, Tottori 683-8503 Japan; 2 Trans Chromosomics Inc., 86 Nishi-cho, Yonago, Tottori 683-8503 Japan; 3 Department of Chromosome Biomedical Engineering, School of Life Science, Faculty of Medicine, Tottori University, 86 Nishi-cho, Yonago, Tottori 683-8503 Japan; 4 Laboratory of Bioengineering, Faculty of Life Sciences, Tokyo University of Pharmacy and Life Sciences, 1432-1 Horinouchi, Hachioji, Tokyo 192-0392 Japan; 5 Department of Chromosome Biomedical Engineering, Integrated Medical Sciences, Graduate School of Medical Sciences, Tottori University, 86 Nishi-cho, Yonago, Tottori 683-8503 Japan; 6 Department of Biomedical Science, Institute of Regenerative Medicine and Biofunction, Graduate School of Medical Sciences, Tottori University, 86 Nishi-cho, Yonago, Tottori 683-8503 Japan; 7 Division of Experimental Pathology, Department of Functional Morphology, Faculty of Medicine, Tottori University, Yonago, Tottori 683‑8503 Japan; 8 Department of Molecular Therapy, National Institute of Neuroscience, National Center of Neurology and Psychiatry (NCNP), Kodaira, Tokyo 187-8502 Japan; 9 Department of Cell Physiology, The Jikei University School of Medicine, 3-25-8, Nishi-shinbashi, Minato-ku, Tokyo, 105-8461 Japan; 10 Department of Chromosome Biomedical Engineering, Institute of Regenerative Medicine and Biofunction, Graduate School of Medical Sciences, Tottori University, 86 Nishi-cho, Yonago, Tottori 683-8503 Japan; 11 Chromosome Engineering Research Group, The Exploratory Research Center on Life and Living Systems (ExCELLS), National Institutes of Natural Sciences, 5-1 Higashiyama, Myodaiji, Okazaki, Aichi 444-8787 Japan

**Keywords:** Genetic engineering, Genetic vectors, Gene therapy

## Abstract

Dystrophin maintains membrane integrity as a sarcolemmal protein. Dystrophin mutations lead to Duchenne muscular dystrophy, an X-linked recessive disorder. Since dystrophin is one of the largest genes consisting of 79 exons in the human genome, delivering a full-length dystrophin using virus vectors is challenging for gene therapy. Human artificial chromosome is a vector that can load megabase-sized genome without any interference from the host chromosome. Chimeric mice carrying a 2.4-Mb human dystrophin gene-loaded human artificial chromosome (DYS-HAC) was previously generated, and dystrophin expression from DYS-HAC was confirmed in skeletal muscles. Here we investigated whether human dystrophin expression from DYS-HAC rescues the muscle phenotypes seen in dystrophin-deficient mice. Human dystrophin was normally expressed in the sarcolemma of skeletal muscle and heart at expected molecular weights, and it ameliorated histological and functional alterations in dystrophin-deficient mice. These results indicate that the 2.4-Mb gene is enough for dystrophin to be correctly transcribed and translated, improving muscular dystrophy. Therefore, this technique using HAC gives insight into developing new treatments and novel humanized Duchenne muscular dystrophy mouse models with human dystrophin gene mutations.

## Introduction

Duchenne muscular dystrophy (DMD) is an X-linked recessive disorder characterized by progressive muscle weakness. It affects 1 every 5000 boys worldwide^1^. Its causative gene is dystrophin, which consists of 79 exons with seven promoters. Dystrophin localized at the cytoplasmic surface of the sarcolemma is linked to glycoproteins, and dystrophin-glycoprotein complexes play a role in stabilizing linkages between intracellular actin cytoskeleton and extracellular matrix^2^. More than 7000 dystrophin mutations were reported, and large mutations, such as deletions and duplications with more than one exon, account for 80% of total mutations, leading to the production of non-functional dystrophin proteins^3^.

Gene therapy using some vectors to deliver genes of interest can compensate for genetic mutations. Adeno-associated virus (AAV) was used to develop treatments for neuromuscular dystrophy due to its capability of systemic gene delivery, high infection efficiency, and large encapsulation capacity^1^. However, because the loading capacity of AAV for a gene and regulatory elements is 5 kb, it is challenging to deliver a full-length dystrophin (14 kb mRNA). To overcome this limitation, some truncated forms called on micro/mini-dystrophin or multiple AAV vector systems were generated to restore dystrophin expression^4^.

Human artificial chromosome (HAC) constructed from a native human chromosome by top-down approach is a vector that can load transgenes of interest, including regulatory elements at the megabase level for physiological gene expression; it is independently maintained without any interference from other gene loci^5^. Previously, chimeric mice with HAC carrying the 2.4-Mb entire human dystrophin gene (DYS-HAC) were generated, and dystrophin expression in the sarcolemma of skeletal muscle was confirmed^6,7^. Moreover, intramuscular and intra-arterial transplantation of corrected mesoangioblasts, blood vessel-associated stem cells derived from mdx mice with DYS-HAC, ameliorated muscular dystrophy phenotypes of mdx/SCID mice, demonstrating the therapeutic potential of DYS-HAC^8^.

*DMD-null* mice lacking the 2.4-Mb dystrophin gene locus were generated by Cre-loxP recombination system^9^. Since the complete absence of dystrophin expression causes severe muscular dystrophy, *DMD-null* mice were thought to be an improved model for functional studies of dystrophin and its isoforms. Here, we generated a transchromosomic (Tc) mouse strain in which DYS-HAC was transmitted through the germline, and we investigated whether human dystrophin from HAC could ameliorate muscular phenotypes observed in *DMD-null* mice.

## Results

### Generation of humanized mice carrying HAC with human dystrophin

**Figure 1:**
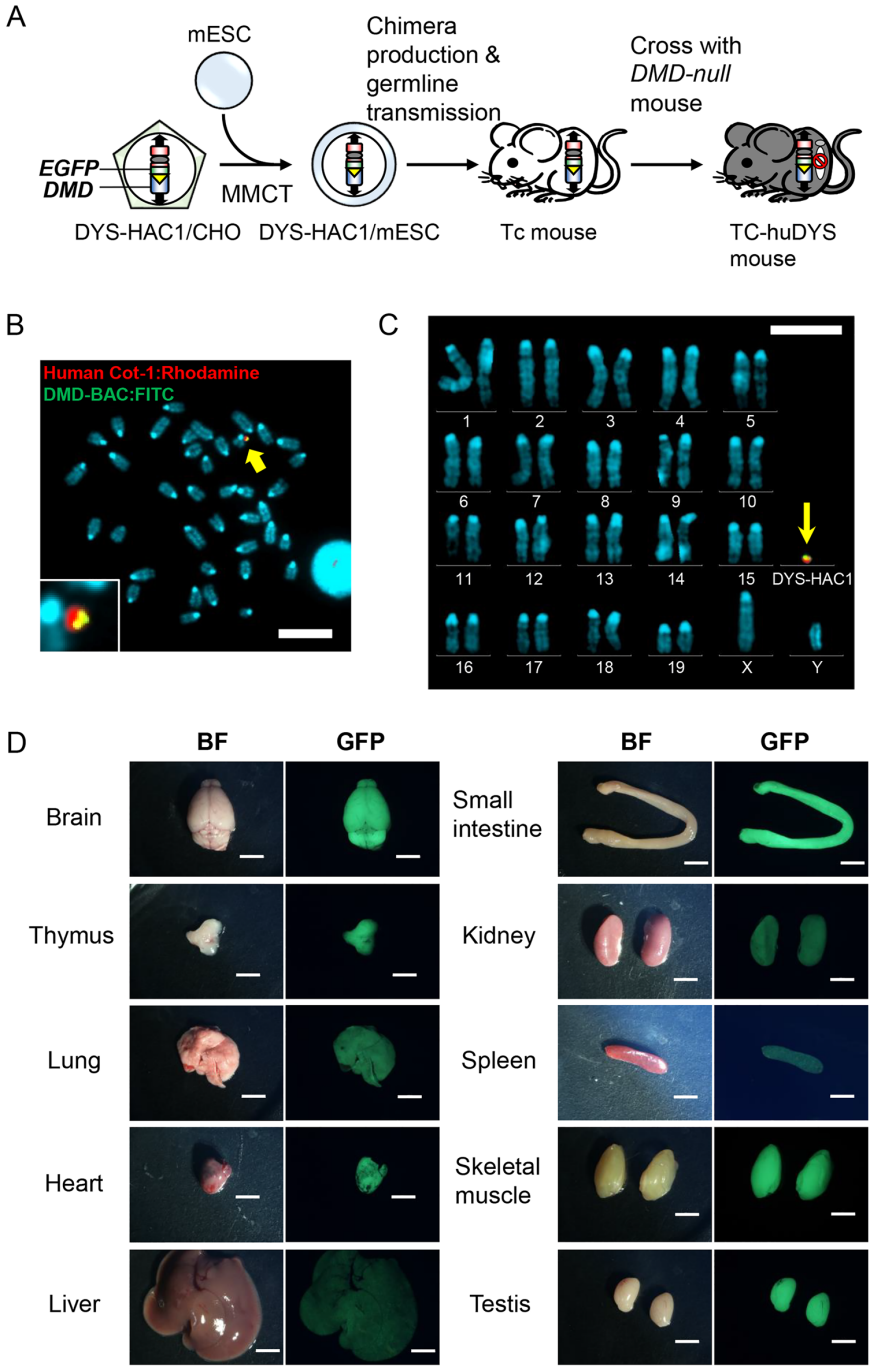
Generation of humanized dystrophin mice. (**A**) Strategy to generate humanized dystrophin mice carrying HAC with human dystrophin (DYS-HAC1). (**B**) Representative FISH image of metaphase spread from microcell hybrid mESCs carrying DYS-HAC1. Red and green indicate HAC and dystrophin gene, respectively. Yellow arrow indicates DYS-HAC1, which is enlarged in the inset. Scale bar: 10 μm. (**C**) Representative karyotyping result of FISH analysis of metaphase spread from lymphocytes in DYS-HAC1; *DMD-null* mice. Red and green indicate HAC and dystrophin gene, respectively. Yellow arrow indicates DYS-HAC1. Scale bar: 10 μm. (**D**) EGFP images of different tissues from DYS-HAC1; *DMD-null* mice. Exposure times for each GFP image were 400 ms for the brain, thymus, lung, heart, liver, small intestine, and kidneys, 2 s for spleen, 40 ms for skeletal muscle, and 200 ms for testis. Scale bar: 5 mm. *BF* bright field.

The HAC containing 2.4-Mb of wild-type (WT) human dystrophin gene (DYS-HAC1) was previously constructed by chromosome engineering technology^6^. To generate humanized dystrophin mice with a *DMD-null* background, DYS-HAC1 was transferred from CHO cells to mouse embryonic stem cells (mESCs) by microcell-mediated chromosome transfer (MMCT) (Fig. 1A). After MMCT, 37 drug resistant clones were obtained, and 25 clones were PCR-positive (Supplementary Table S1). Fluorescence in situ hybridization (FISH) analyses revealed that the obtained microcell hybrid mESCs maintained DYS-HAC1 as an independent chromosome without being integrated into the host genome (Fig. 1B). DYS-HAC1 was transmitted through the germline via chimeric mouse produced from the microcell hybrid mESC line. FISH analysis revealed that DYS-HAC1 was stably maintained in brain, lung, heart, liver, small intestine, kidney, and skeletal muscle (more than 96%), but not thymus (42%) and spleen (57%) (Supplementary Fig. S1). DYS-HAC1 was transmitted to offspring from both WT male and female mice. Genomically humanized dystrophin “TC-huDYS” (DYS-HAC1; *DMD-null*) mice were generated by crossing Tc mice carrying DYS-HAC1 with *DMD-null* mice, and the presence of DYS-HAC1 was confirmed by FISH analysis (Fig. 1C). Since DYS-HAC1 contained CAG-EGFP cassette, EGFP fluorescent signals were observed in various tissues from TC-huDYS mice (Fig. 1D). Germline transmission rate of DYS-HAC1 from *DMD-hetero* female mice was 45.5% (290/638). However, *DMD-null* male mice carrying DYS-HAC1 were sterile. These results suggest that genomically humanized WT dystrophin mice were successfully generated by trans-chromosomic technology with DYS-HAC1.

### Expression of human dystrophin derived from HAC in skeletal and cardiac muscles

To distinguish human dystrophin from mouse dystrophin in skeletal and cardiac muscles, two commercially available anti-dystrophin antibodies, MANDYS106 and ab15277, were used. MANDYS106 and ab15277 recognize human dystrophin in exon 43 and human/mouse dystrophin in exons 77–79, respectively.

**Figure 2:**
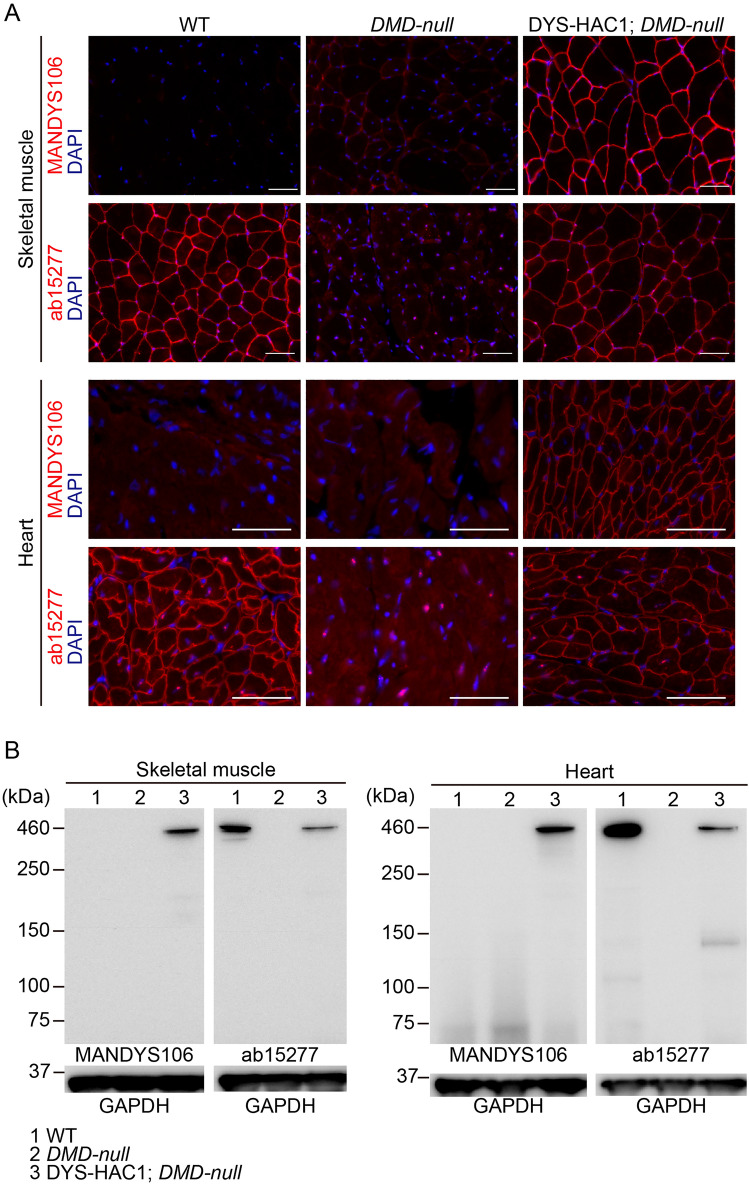
Expression of human dystrophin derived from human artificial chromosome in skeletal muscles and heart. Immunofluorescence (**A**) and western blotting (**B**) for dystrophin in the skeletal muscles and heart of WT, *DMD-null*, and DYS-HAC1; *DMD-null* mice at more than 8 weeks of age. DAPI was used to stain nuclei. GAPDH was used as a loading control. Scale bar is 50 μm. Original blots are presented in Supplementary Fig. S2.

Ab15277 antibody but not MANDYS106 detected mouse dystrophin in the sarcolemma and the expected 427 kDa band (Dp427) in skeletal and cardiac muscles of WT mice by immunofluorescence and western blotting, respectively (Fig. 2A,B, Supplementary Fig. S2). Similar to mouse dystrophin expression in WT mice, both antibodies showed that human dystrophin from DYS-HAC1; *DMD-null* mice was localized in the sarcolemma, and Dp427 was detected in skeletal and cardiac muscles. These results suggest that full-length human dystrophin was normally transcribed, translated, and localized in mice.

Several dystrophin isoforms, such as Dp260, Dp140, Dp71, and Dp40, in non-muscular tissues were reported^10,11^. We investigated human dystrophin isoforms derived from DYS-HAC1 in non-muscular tissues (Supplementary Figs. S3 and S4). Similar to mouse dystrophin expression, Dp71 isoforms were mainly expressed in the brain and testis of DYS-HAC1; *DMD-null* mice. In the liver, both Dp140 and Dp71 isoforms were detected in DYS-HAC1; *DMD-null* mice, while Dp71 was the main isoform of WT mice.

### Function of human dystrophin derived from HAC in skeletal muscles

**Figure 3:**
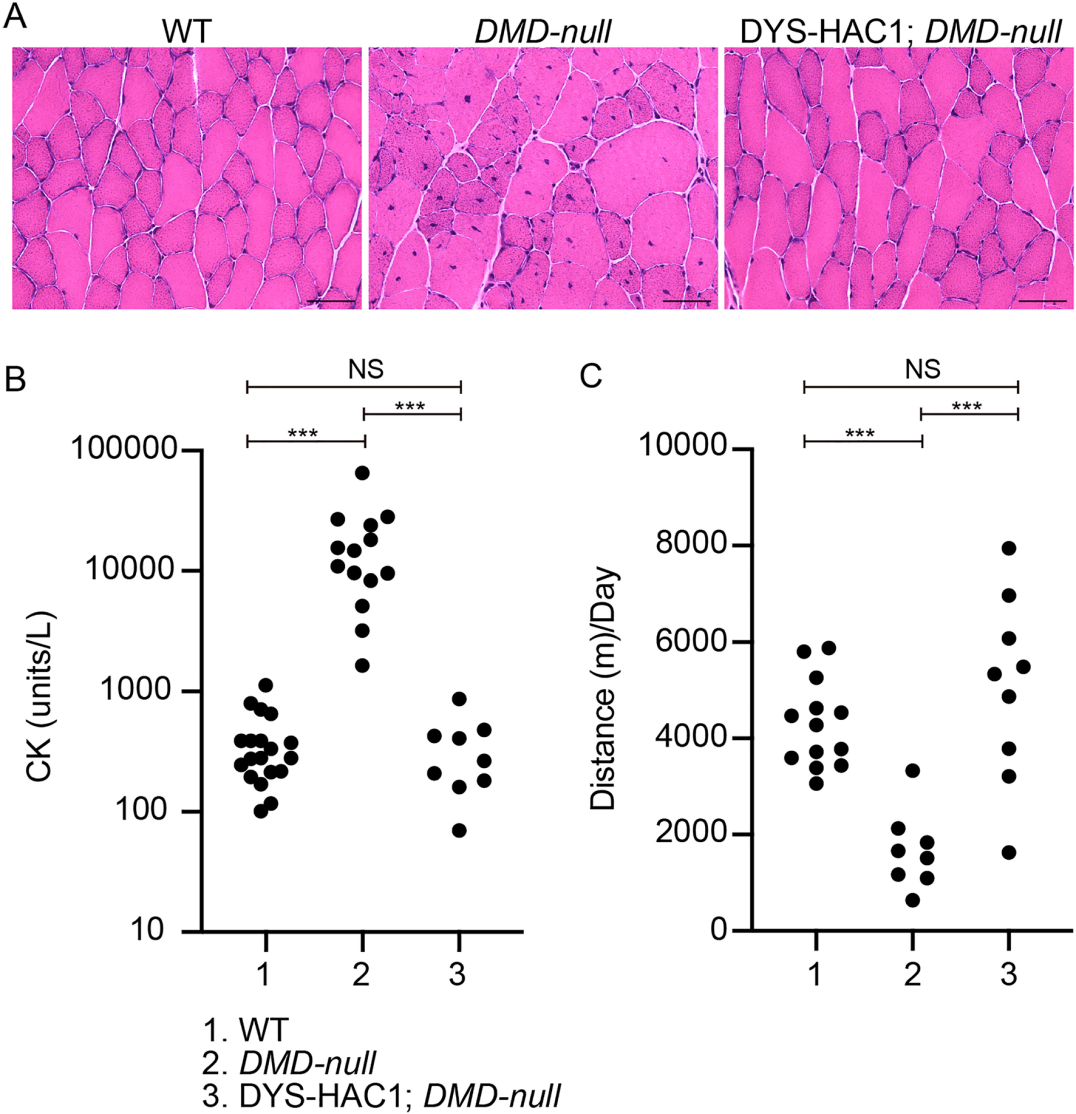
Functional analysis of human dystrophin derived from human artificial chromosome in skeletal muscles. (**A**) H&E staining of skeletal muscles of WT, *DMD-null*, and DYS-HAC1; *DMD-null* mice at more than 8 weeks of age. Scale bar is 50 μm. (**B**) CK activity in WT (n = 19), *DMD-null* (n = 14), and DYS-HAC1; *DMD-null* mice (n = 9) at more than 8 weeks of age. (**C**) Voluntary wheel running in WT (n = 13), *DMD-null* (n = 8), and DYS-HAC1; *DMD-null* mice (n = 9) at more than 8 weeks of age. Tukey’s multiple comparisons test was performed. ***P < 0.001. *NS* nonsignificant.

We investigated whether human dystrophin could rescue muscular phenotypes seen in *DMD-null* mice in terms of histology, physiology, and exercise behavior. Myofibers with internalized nuclei, a hallmark of muscle regeneration, were seen in the gastrocnemius muscle of *DMD-null* mice, while those of DYS-HAC1; *DMD-null* mice were normal similar to those of WT mice (Fig. 3A). Moreover, to confirm the function of human dystrophin systemically, creatine kinase (CK) activity was measured as a marker of muscle damage at more than 8 weeks of age. The CK activity of DYS-HAC1; *DMD-null* mice was lower than that of *DMD-null* mice and was comparable to that of WT mice (Fig. 3B). Furthermore, to study exercise behaviors, voluntary wheel running was performed for three consecutive days. The mean distance per day of DYS-HAC1; *DMD-null* mice was longer than that of *DMD-null* mice and was comparable to that of WT mice (Fig. 3C). These results indicate that human dystrophin derived from DYS-HAC1 compensates for the lack of mouse dystrophin in skeletal and cardiac muscles.

## Discussion

Our previous study showed that human dystrophin in chimeric mice generated from mESCs carrying DYS-HAC was normally expressed and localized in the sarcolemma^6^. As an application of DYS-HAC to stem cell-mediated gene replacement therapy, mesoangioblasts derived from mdx mice carrying DYS-HAC resulted in morphological and functional amelioration of dystrophic phenotype^8^. In this study, we generated DYS-HAC1 mice by germline transmission and investigated the expression and function of human dystrophin in fully dystrophin-deficient mice. The somatic stability of DYS-HAC1 was high in most tissues except spleen and thymus, similar to the previous reports on mice carrying empty HAC with individual variability in HAC stability^12,13^. Consistent with our previous studies, human dystrophin derived from DYS-HAC1 was normally expressed in muscular tissue and improved muscular phenotypes observed in *DMD-null* mice. In addition to these findings, several dystrophin isoforms derived from DYS-HAC1 were also expressed in non-muscular tissues.

Although male sterility in *DMD-null* mice was reported previously^9^, DYS-HAC1 did not improve male sterility in *DMD-null* mice. Considering that a germline transmission of DYS-HAC1 was normally observed in WT male mice carrying DYS-HAC1, there might be a difference in the function of dystrophin in the testis or genetic materials in dystrophin loci between human and mouse. The change in *cis*-regulation around the mouse dystrophin locus in *DMD-null* mouse due to its complete deletion could also be responsible for this phenomenon.

Gene therapies have been considered as attractive approaches to restore the expression and function of dystrophin in DMD patients. Some groups have developed micro/mini-dystrophin using an AAV vector, which ameliorates muscular phenotypes observed in DMD mouse and canine models^4^. In addition to truncated dystrophin forms, a yeast artificial chromosome (YAC) carrying the full-length human dystrophin gene is also engineered^14^. In terms of the expression and function of human dystrophin, both human dystrophin from YAC and HAC worked well, leading to improvement of dystrophic phenotypes seen in DMD model mice. The difference in technique of YAC and HAC is whether a transgene is integrated into the host genome or not, respectively.

Many DMD animal models have been developed for drug discovery and precision medicine^15^. CRISPR/Cas9 gene editing has been applied to develop DMD mouse models carrying mutations seen in patients with DMD^16,17^. Although there is a high homology between the human and mouse dystrophin gene, developing and validating treatments such as antisense oligonucleotides to induce exon skipping targeting the human dystrophin genomic sequences in mice could be attractive. Considering the normal expression and function of human dystrophin derived from HAC in skeletal and cardiac muscles of *DMD-null* mice, the development of DYS-HAC1 containing a mutation based on patients with DMD using genome editing will be applicable to generate a novel humanized DMD mouse model.

In conclusion, Tc mice carrying 2.4-Mb human dystrophin gene were generated using HAC, and it was found that human dystrophin improved the muscle phenotypes of DMD mouse models.

## Methods

### Ethics declarations

This study was approved by the Animal Care and Use Committee of Tottori University (Permit Number: 16-Y-20, 17-Y-28, 19-Y-22, 20-Y-31, 21-Y-26, and 22-Y-36). All experiments were carried out in compliance with the ARRIVE guidelines. All methods were performed in accordance with the relevant guidelines and regulations. Mice were sacrificed by cervical dislocation prior to tissue collection, and all efforts were made to minimize their suffering.

### Cell culture

CHO cells carrying DYS-HAC1 were cultured in a Ham’s F-12 nutrient mixture containing 10% fetal bovine serum and 8 μg/mL BS or 6 μg/mL puromycin. The parental mouse ES cell line, TT2F, and microcell hybrid mESC cell lines containing DYS-HAC1 were cultured as reported previously^18^.

### MMCT

CHO cells carrying DYS-HAC1 were used as a donor for MMCT^19^. Microcell hybrid mESCs containing DYS-HAC1 were selected with 6 μg/mL BS or 0.75 μg/mL puromycin. The transfer of DYS-HAC1 was confirmed by PCR and FISH analyses. PCR primers are listed in Supplementary Table S2.

### FISH analysis

Specimens were prepared from the growing cell culture and tissues according to standard methods. FISH analyses were performed using digoxigenin-labeled (Roche, Basel, Switzerland) human COT-1 DNA and biotin-labeled human dystrophin BAC (RP11-954B16) as described previously^19^. DAPI was used to counterstain chromosomal DNA. FISH images were captured using an AxioImagerZ2 fluorescence microscope (Carl Zeiss, Jena, Germany).

### Generation of chimeric mice

Chimeric mice were produced from microcell hybrid mESCs carrying DYS-HAC1^19^. Microcell hybrid mESCs were injected into 8-cell stage embryos derived from ICR mice (CLEA, Tokyo, Japan) and transferred to pseudopregnant ICR females. Chimeric mice were crossed with ICR males to obtain Tc mice.

### Humanized mice

TK deletion was performed by electroporation (TAKE: Technique for animal knockout system by electroporation)^20^. The target sequence of TK on DYS-HAC1 was 5′-GAG GGC GCA ACG CCG TAC GTC GG-3′.

DYS-HAC1; *DMD-null* mice were generated by crossing Tc mice carrying DYS-HAC1 with *DMD-null* mice. For genotyping, genomic DNA in tails was isolated using a Puregene kit (Qiagen, Hilden, Germany). Genotyping PCR was performed using primers listed in Supplementary Table S2. *DMD-null* mice were provided by the National Center of Neurology and Psychiatry.

### GFP observation in mouse tissues

Photomicrographs of excised mouse tissues were obtained using a microscope (Leica MZ 16F, Leica, Wetzlar, Germany) and NIS Elements D software (Nikon, Tokyo, Japan).

### Sample preparation for histological analysis

Mouse tissues were excised and frozen in liquid nitrogen-cooled isopentane. Transverse sections were made using a cryostat (Leica CM 1950, Leica) and collected onto MAS-GP glass slides (Matsunami, Osaka, Japan) for H&E staining and immunofluorescence and into a 1.5-mL tube for western blotting.

### H&E staining

H&E staining of transverse sections was carried out using a standard method. Photomicrographs were obtained using a microscope (BZ-X810, Keyence, Osaka, Japan).

### Immunofluorescence

The immunofluorescence protocol described previously^21^ was done with slight modifications. Sections (8 μm) were fixed with 4% PFA for 15 min and permeabilized with 0.25% Triton X-100 for 15 min. These sections were incubated with a blocking buffer (1% goat serum and 0.01% Triton X-100) for 30 min, followed by incubation with anti-dystrophin antibodies (ab15277, 1:200, Abcam, Cambridge, UK or MANDYS106, 1:200, Merck, New Jersey, US) at 4 °C overnight and secondary antibodies (Alexa 594 goat anti-rabbit IgG (A32740, 1:500, Thermo Fisher Scientific, Massachusetts, US) or (Alexa 594 goat anti-mouse IgG (A32742, 1:500, Thermo Fisher Scientific)) with DAPI for 1 h. These sections were washed thrice with PBS between each step. For dystrophin staining (MANDYS106), an M.O.M. immunodetection kit (FMK-2201, Vector laboratories, California, US) was used. Fluorescence was obtained using a microscope (BZ-X810, Keyence).

### Western blotting

Previously described western blotting protocol^21^ was done with slight modifications. Approximately 50 10–15-μm thick slices were lysed by a lysis buffer (50 mM Tris–HCl pH 7.4, 150 mM NaCl, and 0.2% Triton X-100), including protease inhibitors (Roche). Lysates were placed on ice for 15 min and spun down at 12,000 rcf at 4 °C for 15 min. After the protein quantification of the supernatant using bicinchoninic acid assay (Thermo Fisher Scientific), the same supernatant concentration was mixed with the equal amount of 2 × Laemmli sample buffer including β-mercaptoethanol, followed by boiling at 95 °C for 5 min. The samples were resolved on 3–8% Tris–Acetate gel (Thermo Fisher Scientific) with Tris–Acetate SDS running buffer at 150 V for 1 h and transferred to a PVDF membrane (Thermo Fisher Scientific) at 20 V for 2.5 h. This was followed by incubation with a blocking buffer (PBS containing 5% dry milk and 0.1% Tween 20) for 1 h, primary antibodies at 4 °C overnight, and secondary antibodies for 1 h. The PVDF membrane was washed three times with PBS containing 0.1% Tween 20 between each step. Signals were detected using an ECL western blotting substrate (Thermo Fisher Scientific) in ImageQuant LAS 4000 mini (GE Healthcare, Illinois, US). For reprobing, the PVDF membrane was incubated in a stripping buffer (Thermo Fisher Scientific) for 15 min. The antibodies used in western blotting were dystrophin (Abcam, ab15277, 1:3000), dystrophin (Merck, MANDYS106, 1:3000), GAPDH (#5174, 1:10,000, Cell Signaling Technology, Massachusetts, US), goat anti-mouse IgG H&L (HRP) (Abcam, ab205719, 1:10,000), and goat anti-rabbit IgG H&L (HRP) (Abcam, ab205718, 1:10,000). HiMark Pre-stained Protein Standard (Thermo Fisher Scientific) and Precision Plus Protein Dual Color Standards (Bio-Rad, California, US) were used as a protein ladder.

### CK activity

The plasma obtained using heparinized micropipettes (Drummond scientific company, Pennsylvania, US) was deposited on a dri-chem slide CPK-PIII (Fujifilm, Tokyo, Japan). CK activity was measured with DRI-Chem 7000 V (Fujifilm).

### Voluntary wheel running

Mice were placed in a general cage attached to a wheel, which was 50 cm in circumference. The rotation number of the wheel was counted by a photo beam sensor for three consecutive days (O’Hara & Co., Ltd, Tokyo, Japan). Logger30 was used as a software.

### Statistical analysis

Tukey’s multiple comparison test was carried out using Prism 8. *P* < 0.05 was considered statistically significant.

## Supplementary Information

Supplementary Information.

## Data Availability

All data generated during this study are included in this published article and its Supplementary Information files.
