# Supplementary material for: Full-length human dystrophin on human artificial chromosome compensates for mouse dystrophin deficiency in a Duchenne muscular dystrophy mouse model
Source: Sci Rep. 2023 Mar 16;13:4360. doi: 10.1038/s41598-023-31481-3 (PMC10020543; doi:10.1038/s41598-023-31481-3)
Supplement: Supplementary file 1 — Supplementary Information. [file 41598_2023_31481_MOESM1_ESM.pdf]

Title

Full-length human dystrophin on human artificial chromosome compensates for mouse dystrophin deficiency in a Duchenne muscular dystrophy mouse model

Authors

Yosuke Hiramuki[1†], Satoshi Abe[2†], Narumi Uno[1,3,4], Kanako Kazuki[1], Shuta Takata[3], Hitomaru Miyamoto[5,6], Haruka Takayama[2], Kayoko Morimoto[2], Shoko Takehara[2], Mitsuhiko Osaki[1,7], Jun Tanihata[8,9], Shin'ichi Takeda[8], Kazuma Tomizuka[4], Mitsuo Oshimura[2], Yasuhiro Kazuki[1,3,5,6,10,11*]

Authors' affiliations

[1] Chromosome Engineering Research Center, Tottori University, 86 Nishi-cho, Yonago, Tottori 683-8503, Japan.

[2]Trans Chromosomics Inc., 86 Nishi-cho, Yonago, Tottori 683-8503, Japan.

[3]Department of Chromosome Biomedical Engineering, School of Life Science, Faculty of Medicine, Tottori University, 86 Nishi-cho, Yonago, Tottori 683-8503, Japan.

[4]Laboratory of Bioengineering, Faculty of Life Sceiences, Tokyo University of Pharmacy and Life Sciences, 1432-1 Horinouchi, Hachiohji, Tokyo 192-0392, Japan.

[5]Department of Chromosome Biomedical Engineering, Integrated Medical Sciences, Graduate School of Medical Sciences, Tottori University, 86 Nishi-cho, Yonago, Tottori 683-8503, Japan.

[6]Department of Biomedical Science, Institute of Regenerative Medicine and Biofunction, Graduate School of Medical Sciences, Tottori University, 86 Nishi-cho, Yonago, Tottori 683-8503, Japan.

[7]Division of Experimental Pathology, Department of Functional Morphology, Faculty of Medicine, Tottori University, Yonago, Tottori 683-8503, Japan.

[8]Department of Molecular Therapy, National Institute of Neuroscience, National Center of Neurology and Psychiatry (NCNP), Kodaira, Tokyo 187-8502, Japan.

[9]Department of Cell Physiology, The Jikei University School of Medicine, 3-25-8, Nishi-shinbashi, Minato-ku, Tokyo, 105-8461, Japan.

[10]Department of Chromosome Biomedical Engineering, Institute of Regenerative Medicine and Biofunction, Graduate School of Medical Sciences, Tottori University, 86 Nishi-cho, Yonago, Tottori 683-8503, Japan.

[11]Chromosome Engineering Research Group, The Exploratory Research Center on Life and Living Systems (ExCELLS), National Institutes of Natural Sciences 5-1 Higashiyama, Myodaiji, Okazaki, Aichi, 444-8787, Japan.

[†] These authors contributed equally to this work.

***Corresponding author:**

Yasuhiro Kazuki, Ph.D

Chromosome Engineering Research Center, Tottori University

86 Nishi-cho, Yonago, Tottori 683-8503, Japan

E-mail: kazuki@tottori-u.ac.jp, Phone: +81-859-38-6219, Fax: +81-859-38-6210

Supplementary Table S1. Genomic PCR results of mESC carrying DYS-HAC1.

Clone No.	DYS 3L/3R	DYS 4L/4R	DYS 5L/5R	DYS 6L/6R	DYS 7L/7R	DYS 8L/8R	TRANS L1/R1
1	+	+	+	-	-	-	+
2	+	+	+	+	+	+	+
3	+	+	+	+	+	+	+
4	+	+	+	+	+	+	+
5	+	+	+	+	+	+	+
6	-	-	-	-	-	-	-
7	+	+	+	+	+	+	+
8	+	+	+	+	+	+	+
9	+	+	+	+	+	+	+
10	+	+	+	+	+	+	+
11	+	+	+	+	+	+	+
12	+	+	+	+	+	+	+
13	+	-	+	+	+	+	+
14	+	+	+	+	+	+	+
15	+	+	+	+	+	+	+
16	+	+	+	+	+	+	+
17	+	+	+	+	+	-	+
18	+	+	+	+	+	+	+
19	-	-	-	-	-	-	+
20	+	+	+	+	+	+	+
21	+	+	+	+	+	+	+
22	+	+	+	+	+	+	+
23	+	-	+	+	+	+	+
24	+	-	+	+	+	+	+
25	+	+	+	+	+	+	+
26	+	+	+	+	+	+	+
27	+	+	+	+	+	+	+
28	+	+	+	+	+	+	+
29	+	+	+	+	+	+	+
30	-	-	-	-	+	+	+
31	+	+	+	-	-	-	-
32	+	+	+	+	+	+	+
33	-	-	-	-	-	+	-
34	+	+	-	-	-	-	-
35	+	+	+	+	+	+	+
36	+	+	+	+	+	+	+
37	-	-	-	-	+	+	+

Supplementary Table S2. Primer list for genomic PCR analysis.

Gene or aim	Primer name (forward)	Forward primer (5'-3')	Primer name (reverse)	Reverse primer (5'-3')	Product size
hDystrophin	DYS 3L	AACAACTGAACAGCCGGTGGA	DYS 3R	GGGGTGGTGGGTTGGATTTT	163 bp
hDystrophin	DYS 4L	GCAAGAGCAACAAAGTGGCCTA	DYS 4R	AGCTTCTTCCAGCGTCCCTCA	128 bp
hDystrophin	DYS 5L	ACCTTCAGAACCGGAGGCAAC	DYS 5R	AGGGACCCTCCTTCCATGACTC	132 bp
hDystrophin	DYS 6L	TGGAACGCATTTTGGGTTGTT	DYS 6R	AAAACAATGCGCTGCCTCAAA	170 bp
hDystrophin	DYS 7L	TTTGCATCCTTTTGGCGTGAT	DYS 7R	AAACTCAAGCCTGCCCCACTC	151 bp
hDystrophin	DYS 8L	GCTGCTAGCAATGCCACGATT	DYS 8R	GGATGGGCTGGGAATCCATAG	155 bp
DYS-HAC1 detection	TRANS L1	TGGAGGCCATAAACAAGAAGAC	TRANS R1	CCCCTTGACCCAGAAATTCCA	408 bp
Dmd null allele detection	EGFP L	CCTGAAGTTCATCTGCACCA	3mDys Rv	TGTTCTGGTTGAAACTAAGGCTCCAATG	＞4.0 kbp
Dmd null allele detection	mDys KO up Fw	TTCCCCAACAGAGAGGCATGCTTGGAAGTTTTACA	mDys KO dwn Rv	ACCTGCCTCCTAGGATGATGTGGTGAGGAGTCAAC	4.0 kbp
mDystrophin	Dystrophin Fw	TGGGCAAGAGTGAATTTTCC	Dystrophin Rv	ACCACCCACTTCAGGTTGAG	437 bp

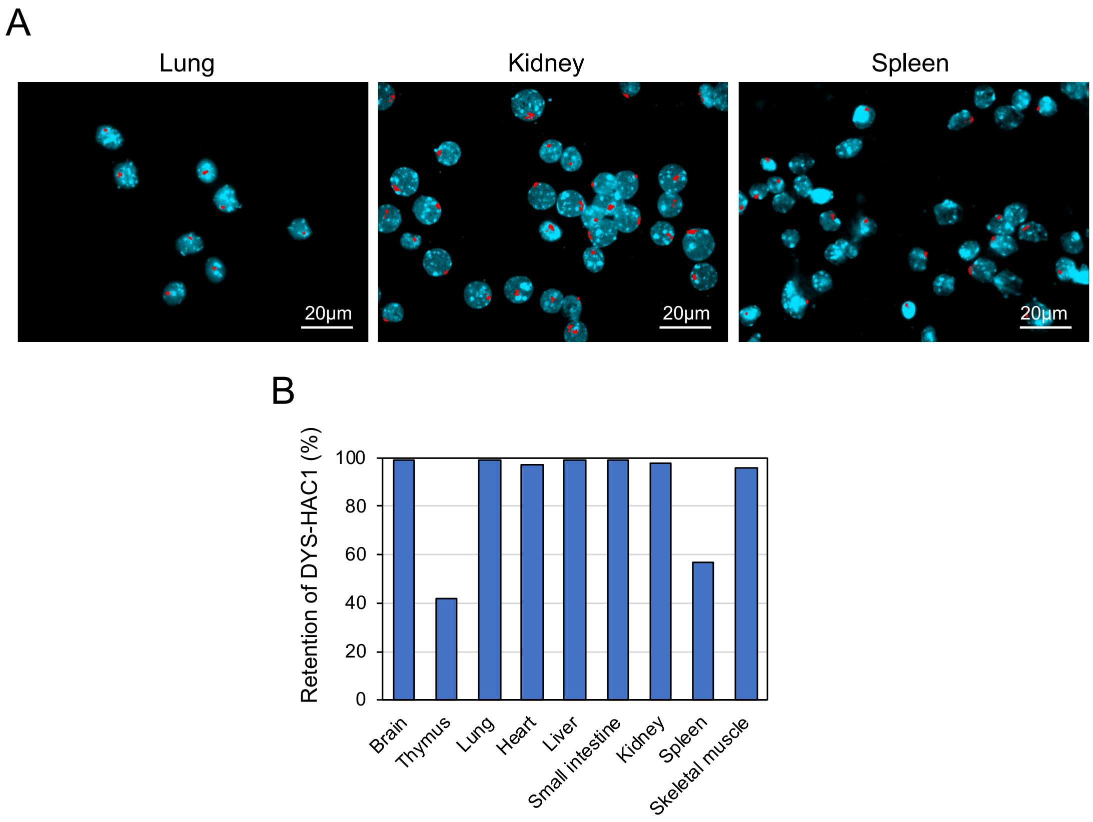

Supplementary Figure S1. Retention of DYS-HAC1 in various tissues of Tc mouse.

(A) Representative FISH images of isolated cells from tissues of Tc mouse carrying DYS-HAC1. DYS-HAC1 is detected in red. (B) Retention rate of DYS-HAC1 in various tissues of Tc mouse.

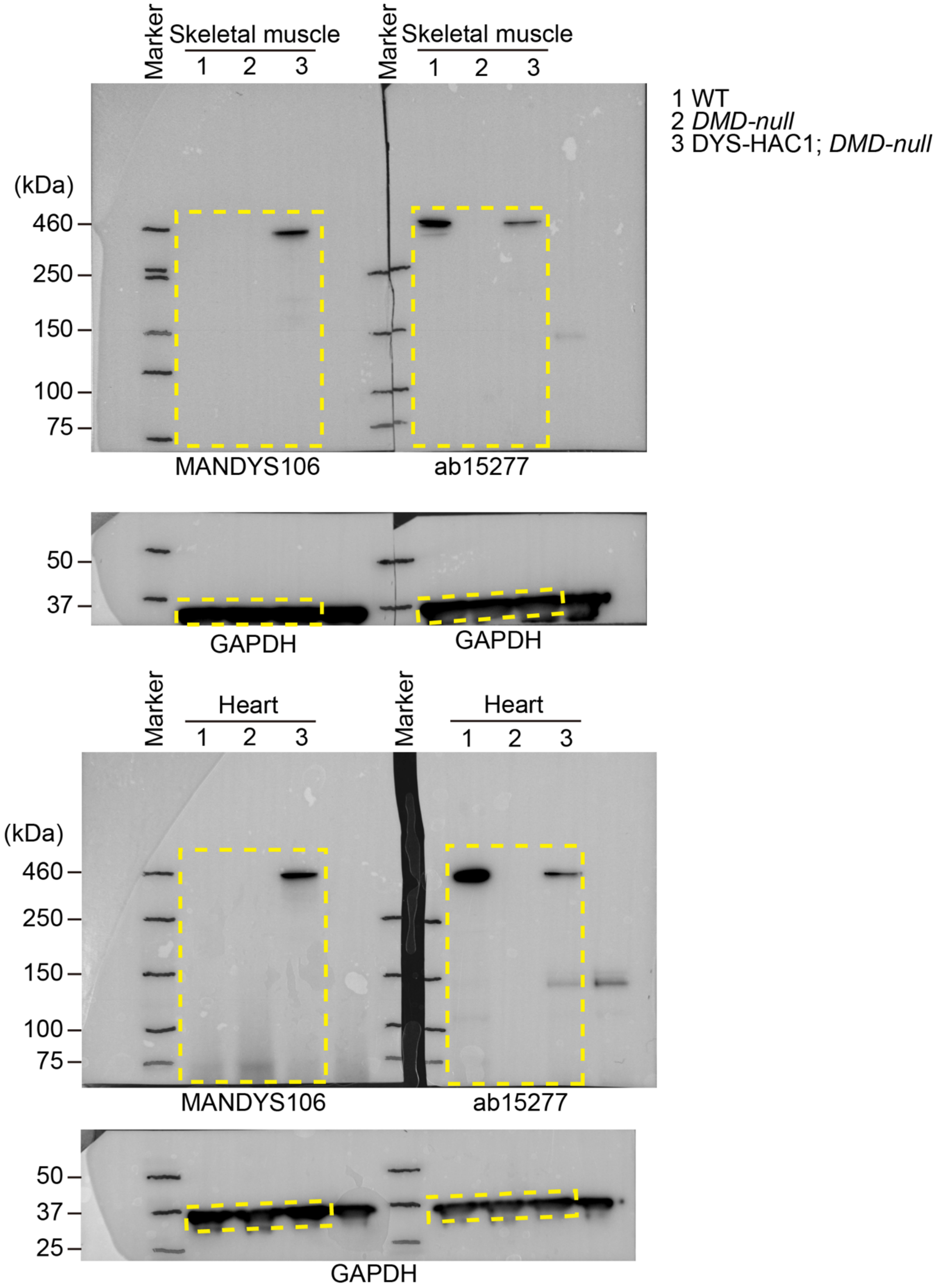

Supplementary Figure S2. Original blots for Figure 2.

Yellow dash lines indicate the clipped area in Figure 2.

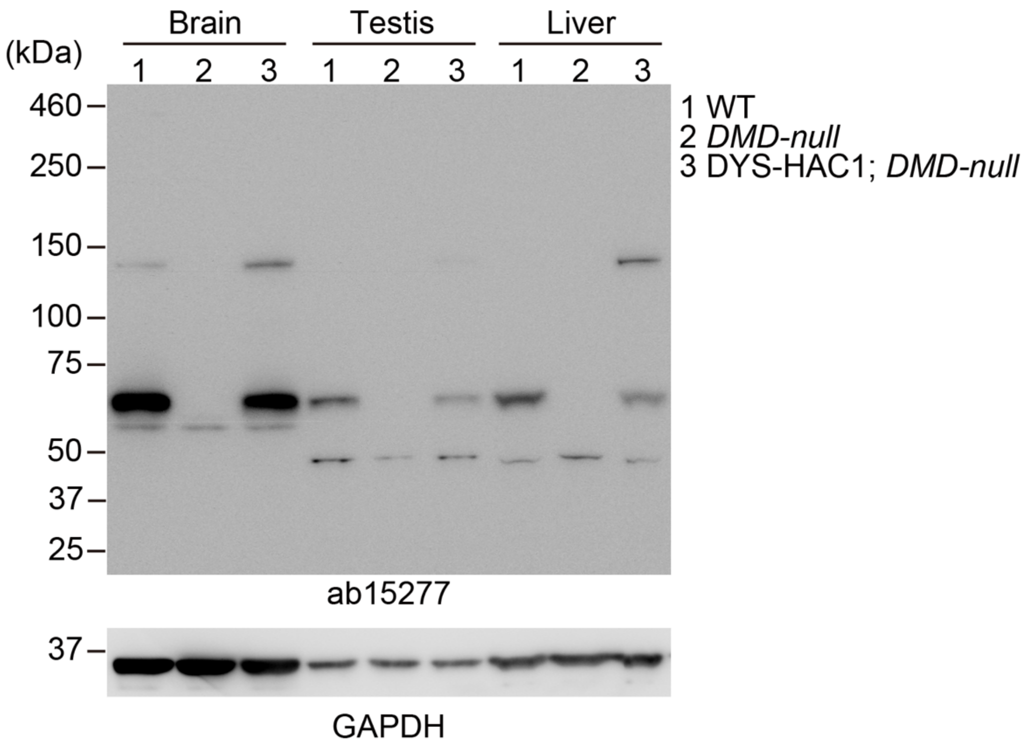

Supplementary Figure S3. Expression of human dystrophin derived from human artificial chromosome in non-muscular tissues.

Western blotting for dystrophin in the brain, testis, and liver of WT, *DMD-null*, and DYS-HAC1; *DMD-null* mice at more than 8 weeks of age. GAPDH was used as a loading control. A PVDF membrane was reprobed with GAPDH antibody after stripping. Original blots are presented in Supplementary Figure S4.

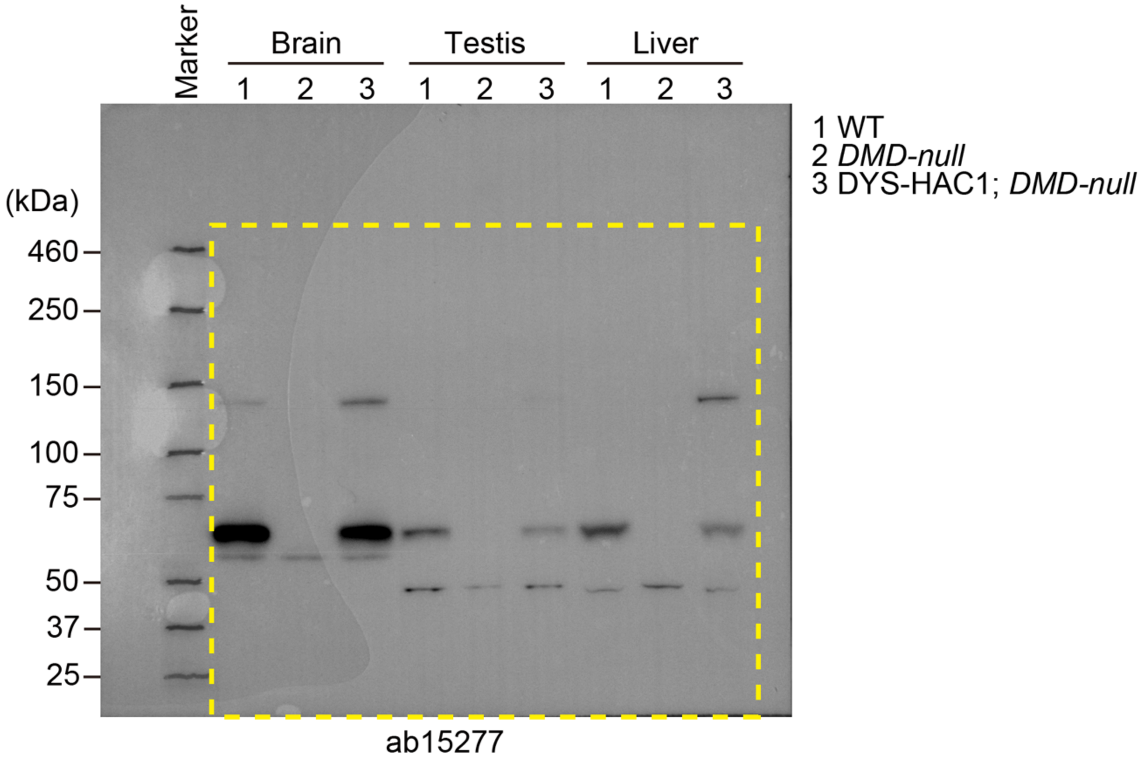

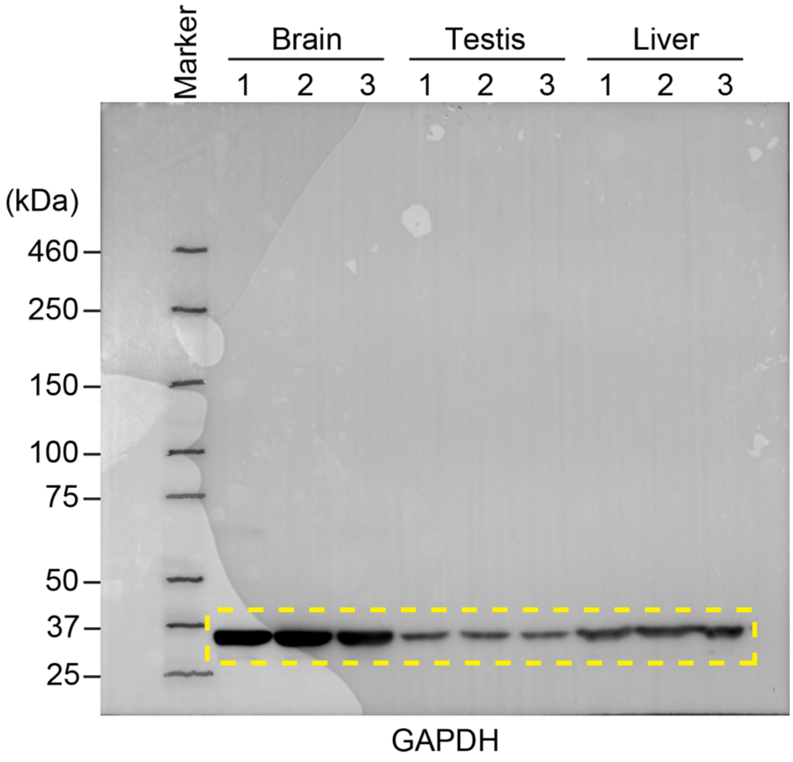

Supplementary Figure S4. Original blots for Supplementary Figure S3.

Yellow dash lines indicate the clipped area in Supplementary Figure S3.
